# Implementation research can enhance health research systems and reduce dependency on external aid

**DOI:** 10.1371/journal.pgph.0006144

**Published:** 2026-03-30

**Authors:** Catherine Chunda-Liyoka, Justin Pulford, Bernard Appiah, Susie Crossman, Motto Nganda, Obiageli Nnodu, Alex Osei-Akoto, Frederic Piel, Tara Tancred, Imelda Bates

**Affiliations:** 1 University Teaching Hospitals-Children’s Hospital, Lusaka, Zambia; 2 Centre for Capacity Research, Liverpool School of Tropical Medicine, Liverpool, United Kingdom; 3 Syracuse University, Syracuse, New York, United States of America; 4 Department of Haematology and Blood Transfusion/Centre of Excellence for Sickle Cell Disease Research and Training (CESRTA), University of Abuja, Abuja, Nigeria; 5 Komfo Anokye Teaching Hospital/Kwame Nkrumah University of Science and Technology, Kumasi, Ghana; 6 Imperial College, London, United Kingdom; The University of British Columbia, CANADA

## Abstract

Implementation research is essential for moving research findings into everyday practice. It is intentionally geared towards local ownership and sustainability, and contributes to more effective health policies, improved service delivery, better resource allocation and strengthened health research systems. By engaging with non-specialist researchers who have in-depth understanding of the context in which interventions occur, implementation research utilises existing capacity and ingenuity for localised problem-solving. Although it is now changing, the traditional global health research paradigm has been of extractive research which undermined local knowledge and expertise and perpetuated asymmetrical research relationships. The failure of global research partnerships to focus on systems strengthening has hindered the ability of poorer countries to compete internationally for research funds, meaning that countries with the weakest health research systems have been hardest hit by the recent shrinking of international health research and programme funds. The gap between decolonial discourse and action is wide. The ‘research for development’ community is significantly under-exploiting the potential of stronger implementation research capability in the world’s poorest countries to narrow this gap. A deliberate and sustained shift toward strengthening research systems with a greater focus on implementation research would be an effective way to promote genuine research partnerships. It could also contribute to reducing external dependency on programme delivery and agenda-setting and promote more equitable global health outcomes.

## The need for implementation research

Implementation research helps us to understand what, why, and how interventions work in “real world” settings and to test approaches to improve them [1]. It is essential for moving research findings and evidence into everyday practice and contributes to more effective policies and programmes. Within health research, this means adapting and embedding contextualised interventions within health systems and making sure they are fit for purpose [2]. Implementation research can make a major contribution to filling the ‘know–do’ gap. This gap is the failure to act on evidence and knowledge from research to improve health outcomes primarily due to lack of timely, quality and relevant research evidence and insufficient collaboration with policymakers [3]. Implementation research is part of the final common pathway by which new interventions are tested and operationalised in real world settings and strategies developed for their scale-up and sustainability.

Although implementation research can be applied to policies, programmes and practices, our focus in this paper is on how diverse actors can work together to implement sustainable evidence-based health interventions in resource-limited settings. Implementation research differs from other research paradigms in that it is driven by the needs of local stakeholders and the realities of the real world - it supports the contextualisation of interventions to enable their routine use, scale-up, and sustainability, rather than the imposition of interventions that have been developed and assessed external to the context [4]. It is complementary to other types of health research such as policy and systems research and operational research, which focus on how systems and different actors organise themselves to contribute to policy outcomes [5].

Implementation research is multidisciplinary and can adopt qualitative, quantitative, and mixed methods to explore outcomes such as acceptability, appropriateness, feasibility, implementation cost, coverage, and sustainability of an innovation such as a health intervention or an implementation strategy. Implementation research relies on engagement of diverse, local stakeholders. It builds on existing capacity and ingenuity for localised problem-solving, and creativeness in resource mobilisation by engaging non-specialist researchers such as patients, healthcare staff, policy makers, media specialists and others in the implementation research process. This potentially strengthens the health research system by increasing the national health research workforce and health research literacy and by connecting research to practice and policy.

Implementation research promotes engagement with cross-sectoral stakeholders

“ *And it [implementation research] also helps you reach out more to the world rather than just reaching out to your patients”* [6]. [00:06:27]*

*The quotes in this article are experiential and provided by colleagues in sub-Saharan Africa who contributed to a 3-part podcast describing their experiences of being involved in their first implementation research project (on sickle cell disease). *[xx] denotes the quote’s location in the podcast* [6*–*9].

Implementation research also inherently contributes to strengthening the broader health system through the activities of individual implementation research projects and by the skillsets and processes that such projects can embed in the health system.

Implementation research can contribute to sustainable health system strengthening

*Because when you collaborate with the team from the beginning to the end, you are building a system of sustained interventions where teams and individuals pick up or embed key components of that capacity-strengthening within your routine systems. So, really bringing home that co-creation, moving together, think big for sustainability and longevity throughout the process* [8]. [00:21:39]

Implementation research can address complex challenges within health systems and inform policy-making, optimise resource allocation and improve service delivery [4]. Through the process of different actors ‘learning by doing’ and adapting innovations to the health system, positive outcomes may extend beyond those anticipated in the original implementation research project [10].

Implementation research practitioners develop core competencies including team-building, problem identification and prioritisation, flexible strategic planning for activities, scale-up and sustainability, and continuous learning [11]. Developing these competencies requires first hand involvement in projects that use implementation research approaches. The skills and knowledge that these individuals acquire can be applied across research disciplines and in diverse contexts and are essential for supporting national research leaders to identify and solve nations’ own health problems.

Compared to many other types of research, implementation research is intentionally geared toward local ownership and sustainability, arguably creating more impactful use of research funding and reducing dependency on external agenda-setting and programme delivery. These aspects of implementation research make it invaluable in the current geopolitical context. Shrinking research funds for international development are creating an imperative to accelerate local ownership and to redress inequities in the ability of low- and middle-income countries (LMIC) by reducing reliance on external funding to support local health systems strengthening. Countries with the weakest health systems have been among those hardest hit by funding cuts. This is one of the consequences of the colonial heritage of extractive research which undermined local knowledge and expertise and perpetuated asymmetrical research relationships [12].

High-quality and impactful locally appropriate research can improve development outcomes [13]. Health research, particularly implementation research, is therefore an essential component of any health system. Some funders such as the Canadian Institutes of Health Research and the Global Fund have a long tradition of supporting implementation science and direct national funding to enable a greater degree of local ownership. Strengthening capacity to carry out implementation research - across its processes and its practitioners - should be at the centre of all research for development. The implementation research process involves collaborating with health systems stakeholders to, for example, jointly identify local problems or barriers to operationalisation of evidence-based practices and test ways to overcome them. Solutions created through these processes can be locally embedded and owned. Implementation research practitioners typically include professional researchers, but also beneficiaries of the research, healthcare staff and policy makers, including those who are research-naïve.

Implementation research is a mechanism for accelerating dissemination and uptake of research findings

*We have formed between the researchers, journalists, and also policy makers, a forum such that if research findings come out, it’ll be easy to be able to be captured by journalists and given out to the public, as well as the policy makers also using that for policy change and all that. And these are all in the end going to help patients* [7]. [00:18:26]

Implementation research is therefore a potentially powerful and low-cost mechanism for promoting greater autonomy for researchers and institutions in LMIC, by reducing their reliance on external funders and expertise, while simultaneously strengthening national health research systems. However, it is underutilised, partly because it may not be well understood or formalised, and because it can entail being comfortable with uncertainty or be perceived as lacking in legitimacy [14].

The Centre for Capacity Research is a global leader in the science of health research systems strengthening and have a long history of research partnerships with colleagues in LMIC. Using, and strengthening national capacity for, implementation research is core to much of our practice since we need to understand how research can impact on health systems and how context moderates that impact at different levels of research systems. From our Centre’s perspective it appears that the ‘research for development’ community is significantly under-exploiting the potential of stronger implementation research capability in LMIC to promote more resilient health systems.

## Current funding models do not address inequities

Although implementation research is gaining traction, it has received relatively little purposeful investment. As a result, its potential to strengthen both health systems and health research systems is under-recognised and under-utilised.

Implementation research is subject to some constraints. It has to operate within existing power structures and can therefore be hampered by donor-driven funding models and other external agencies’ strategies. Some of these agencies’ approaches may unintentionally mitigate against equity and local ownership. For example, funders may require the lead organisation to be based in a high-income country institution, which then disburses project funds to partners in less well-resourced countries. This disempowers these partners and makes it difficult for them to gain experience as financial leads for projects. Funders are increasingly requiring research - even research for development - to have substantial and tangible benefits for the donors’ own country, which can distort project goals and constrains the amount of investment and capacity strengthening that can be built in for partners in lower-income countries. Although implementation research is by definition responsive to the stakeholders’ needs, the requirement to define project outputs and pathways to impact at the proposal stage reduces flexibility and potentially curtails a project’s ability to respond to new challenges that emerge during the project lifetime.

Implementation research involves inputs by multiple stakeholders including non-academics and the current paradigm for academic authorship does not adequately reflect the diverse contributions to research papers. In some countries, the status of ‘first’ or ‘last’ author contributes to institutional rankings that are linked to government funding. This, and the unwillingness of some journals to list multiple authors or recognising joint lead authorship, can lead to skewing of research benefits to collaborators from high-income settings.

Between 2016–2021, the UK government and Wellcome collectively spent £873 million on dedicated initiatives to strengthen research capacity in LMIC with a further £1.2 billion expended on research activities with a capacity strengthening component [15]. Nevertheless, overall inequities in research capacity remain with high-income countries having 4,301 researchers per million inhabitants compared to 45 per million in low-income countries, and 1,227 versus 25 scientific publications per million inhabitants [16]. Traditionally the largest proportion of overall spend on strengthening research capacity in LMIC has been via research partnerships. There has been a strong bias towards investing in individuals (for example PhD scholarships) rather than the institutions or broader systems in which they work.

The lack of long-term investment in research systems in LMIC has perpetuated a cycle in which limited research capacity in the South drives dependency on Northern partners and funders. This dependency means that Northern-focused scientific agendas may take precedence over LMIC’s national research priorities and fail to address systemic weaknesses in LMIC health systems [17]. Consequently, despite receiving large investments for strengthening research capacity, the underlying inequities and dependency have not been addressed.

## A period of change

“Research excellence, investment in research and global collaboration are the bedrock for advancing health through science. And good health and health security are fundamental for the strong economies and societies all nations desire” [18]. Nevertheless, declining overseas aid budgets exacerbated by the more recent “abrupt” and “sweeping suspension of US foreign aid” have impacted critical global collaborations that were providing health care, shelter and food security for people in vulnerable situations [19]. By February 2025 the US’s NIH, which is the world’s largest public funder of biomedical research, had had its budget of US$1.5 billion frozen, putting 16,000 grant applications in limbo and jeopardising 300,000 researchers’ work. The US CDC’s workforce has been reduced by 10% which affects it ability to investigate and respond rapidly to public health threats globally, and cripples programmes for vaccinations, mothers and children, infectious disease prevention and water and sanitation [20].

Europe too is cutting its overseas aid budgets [21]. The UK and Germany account for around 12–15% of global humanitarian assistance and planned reductions in their budgets will drastically affect life-saving projects worldwide [22]. Organisations dependent on this funding potentially face staff reductions and program closures which will inevitably hinder progress towards the 2030 Sustainable Development Goals, particularly in health, education, gender equality, poverty reduction and food security.

Despite the billions of US$ spent on ‘North-to-South’ funding for strengthening research capacity in LMIC, the detrimental impact of these cuts, especially on countries with weak health systems, is a stark reminder of the dependency of LMIC on external agencies. It also highlights the major global inequities that exist in LMIC’s ability to sustain health research and health programmes. These inequities will be exacerbated by climate change since LMIC populations are particularly vulnerable to the health effects of climate change due to their geographical location, low socioeconomic status and weak healthcare infrastructure [23].

It is widely recognised that knowledge hierarchies and asymmetrical dynamics in health research between high-income countries and LMIC can hinder research impact [24]. Consequently the 2024 World Health Summit in Berlin has produced six key strategies to redress the balance and promote more equitable global health outcomes including strengthening LMIC’s research systems, aligning research with national priorities, enabling LMIC institutions to manage projects independently and supporting LMIC-led, context-relevant research initiatives.

Implementation research, with its focus on priorities that are meaningful to local stakeholders, generating rich, contextually-dependent insights and innovations, is an effective mechanism to contribute to levelling the playing field in global production of knowledge. Furthermore, implementation research helps to align sectors and stakeholders to avoid duplication and to ensure a comprehensive approach to each challenge [25]. The gap between decolonial discourse and action is wide, but sustained investment in strengthening research systems including through the more equal types of partnerships that are at the heart of implementation research approaches, can help to achieve genuine equity [26].

## Creating new opportunities

**Fig 1:**
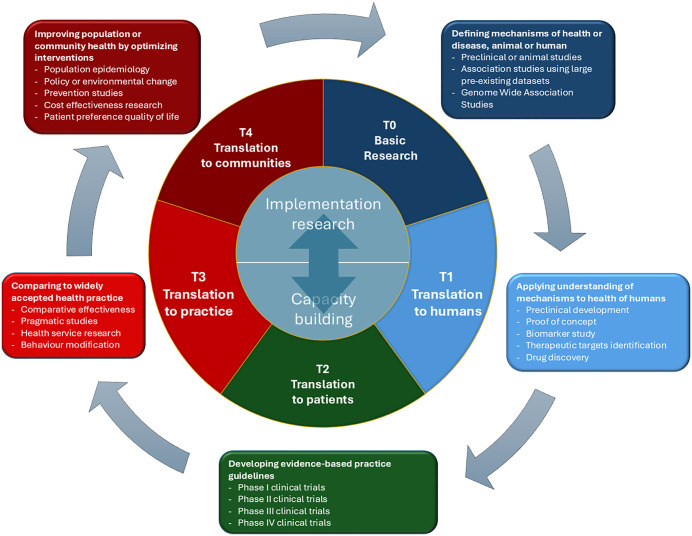
Clinical and translational research classification.

It could be argued that governments in LMIC should be investing more in their own research systems and overall this has been happening. In 2019 the world’s governments funding for basic research and product development on neglected diseases peaked at 81% but dropped to 63% ($2,622 million) in 2023 partly due to a drop in US government funding. However, public funding from LMIC increased by over 20% (totalled $113m in 2023) which was 16% above its long-term average. This was predominantly due to an increase in India’s spending though this was nowhere near enough to offset the loss of investment from high-income countries [27]. Compared to other funders, LMIC governments are more heavily focused on basic research and spend only 23% of public money on the later stages of the health research pathway (i.e., the translation of findings into practice and communities) (Fig 1) [28]. There may therefore be some scope for shifting LMIC’s own public investments towards more implementation research.

Implementation research can focus on problem-solving and centring patients

*So, for me, that [implementation research] is something I find really interesting. It almost like answers all the questions. It’s a roundabout way of answering all the potential problems that patients may have. In implementation research or in the cycles that we’re following, we are actually trying different ways of solving a problem so that if this doesn’t work, you can abandon it and start something else. And you are actually doing that not just as clinicians, sitting up high there and we have the solutions now, but actually involving the very people that you’ve identified the problem in getting them on board to actually find out what could be done different so that things are different for them* [7]. [00:23:59]

The evaluation of implementation research can be complex and realist evaluation may be helpful because it can identify feedback loops and intended and unintended outcomes [29], which may be under-investigated through other methods. Its use within global health research is increasing because it seeks to understand what aspects of an intervention/implementation strategy worked, for whom, and under which condition. It centres the role of context and aims to understand the mechanisms by which an intervention or implementation strategy embeds and produces desired impacts [30].

## Conclusions

We contend that implementation research is underutilised as an approach to potentially increase empowerment and equity in global health. It can help to overcome the know-do gap, driving the embedding and sustainment of critically important practices. It can accelerate uptake of research findings and bring innovations to scale to ensure impact on practices and communities. By joining forces through implementation research, government actors, health professionals, academic institutions, and communities can be instrumental in strengthening systems for health and health research, improving quality of care, enhancing equity and efficiency and empowering beneficiaries [4,31]. Such a transformation depends on shifts in power through equitable partnerships, sustained investment and participatory approaches to learning which are all characteristics of implementation research.

Although it has been described as “a key to addressing challenges and bottlenecks in health systems” [32] implementation research alone cannot overcome entrenched structural challenges to achieve sustainable change. It takes place in the context of health systems and policies so its impact can be hindered by poor engagement of stakeholders including the public, lack of political prioritisation or demand, insufficient or inappropriate contextualisation and limited availability of human and financial resources [33]. It complements but does not replace related research on, for example, health systems or policy and programme planning.

Implementation research is a relatively young field but growing in importance. Implementation research practitioners and those that support implementation research, learn to connect, understand, inspire, enable, and transform [1]. In doing so, they gain confidence and competence in contextualising and scaling up successful innovations, in embedding them in local and national systems and in influencing policy and practice. These are essential attributes for strengthening national research capability and for improving and sustaining strong health systems.

Implementation research promotes equity and local ownership

*You have to develop that skill to be able to think, to be able to do proposals, to be able to generate ideas and to be, you know, a core contributor in that process instead of just being at the receiving end of it. So that equity is very important, and it can only be done intentionally* [7]. [00:09:23]

The traditional model - in which funding to support research partnerships for better health outcomes in LMIC comes from the more ‘wealthy’ countries - is diminishing. Drastic and rapid cuts in wealthy countries’ overseas aid budgets have jeopardised programmes for the most vulnerable people in some of the world’s poorest countries. Recent cuts in ‘north to south’ health research funding flows have exposed the major inequities in the ability of LMIC to sustain health research and health programmes that exist. Breaking the ‘aid dependency’ cycle requires a deliberate re-balancing with a shift toward strengthening research systems in LMIC. Implementation research is an underutilised potential mechanism for reducing dependency on external financial aid and improving resilience in the face of dwindling external support [33]. Implementation research is a critical tool within the confines of real resource constraints. It works within existing structures and realities, not against them, and can therefore bring about feasible change. The use of implementation science by LMIC researchers can lead to meaningful reductions in dependence on external aid and expertise as researchers learn to design and conduct research into existing local health problems and design interventions to alleviate them. Stronger research systems would promote resilience and give LMIC greater autonomy to determine their own research priorities and in deciding who to partner with. Sustained investment in research systems strengthening and a greater focus on implementation research would be an effective way to promote more genuine equity and enable Southern researchers and institutions to engage with Northern partners on a more equal footing.

Of particular importance are the skills and knowledge of individuals and institutions in designing, conducting and managing their own implementation research, which is growing rapidly in LMIC. Implementation research explicitly requires collaboration with populations that will be implementing or affected by an intervention and depends on having an in-depth understanding of the context in which implementation occurs. This includes the cultural, social, political and physical environments in which health systems and their institutions exist. LMIC-based implementation researchers therefore have enormous advantages over external researchers, given their extensive knowledge of local systems and contexts. Recognising and enhancing their strengths, alongside creating institutional environments and systems where implementation research can thrive and flourish, will go a long way towards promoting the resilience of health research systems and equity in health research partnerships.
